# A longitudinal study on artificial intelligence adoption: understanding the drivers of ChatGPT usage behavior change in higher education

**DOI:** 10.3389/frai.2023.1324398

**Published:** 2024-01-05

**Authors:** Athanasios Polyportis

**Affiliations:** Department of Biotechnology, Faculty of Applied Sciences, Delft University of Technology, Delft, Netherlands

**Keywords:** chatbot in higher education, ChatGPT, artificial intelligence adoption, longitudinal survey, emotional creepiness, Perceived Behavioral Control, trust in artificial intelligence, student behavior change

## Abstract

As the field of artificial intelligence (AI) continues to progress, the use of AI-powered chatbots, such as ChatGPT, in higher education settings has gained significant attention. This paper addresses a well-defined problem pertaining to the critical need for a comprehensive examination of students' ChatGPT adoption in higher education. To examine such adoption, it is imperative to focus on measuring actual user behavior. While measuring students' ChatGPT usage behavior at a specific point in time can be valuable, a more holistic approach is necessary to understand the temporal dynamics of AI adoption. To address this need, a longitudinal survey was conducted, examining how students' ChatGPT usage behavior changes over time among students, and unveiling the drivers of such behavior change. The empirical examination of 222 Dutch higher education students revealed a significant decline in students' ChatGPT usage behavior over an 8 month period. This period was defined by two distinct data collection phases: the initial phase (T1) and a follow-up phase conducted 8 months later (T2). Furthermore, the results demonstrate that changes in trust, emotional creepiness, and Perceived Behavioral Control significantly predicted the observed change in usage behavior. The findings of this research carry significant academic and managerial implications, as they advance our comprehension of the temporal aspects of AI adoption in higher education. The findings also provide actionable guidance for AI developers and educational institutions seeking to optimize student engagement with AI technologies.

## 1 Introduction

Recent advances in artificial intelligence (AI) have ushered in a new era in which AI-powered technologies are becoming increasingly integrated into various aspects of society. The emerging prevalence of AI-driven chatbots, such as ChatGPT (OpenAI, 2022), has particularly intrigued researchers, institutions, and society at large, due to their offering of novel opportunities in the fields of communication (Zhou et al., 2023), user satisfaction (Rapp et al., 2021), social interactions (Skjuve et al., 2022), healthcare and medicine (Pernencar et al., 2022; Dave et al., 2023) and notably, educational transformations (Wollny et al., 2021; Lo, 2023). In the realm of higher education, the potential of AI chatbots to assist students, provide information, and support learning processes has become evident (Neumann et al., 2021; Dwivedi et al., 2023). However, as higher education institutions increasingly examine how to incorporate AI chatbots in their curricula, understanding students' adoption and usage behavior has become a matter of significant academic interest and practical relevance.

In this context, the examination of students' behavioral intentions and attitudes toward using AI chatbots has been a primary focus. However, the measure of attitudes and intentions alone does not provide a comprehensive understanding of AI adoption dynamics. To gain a deeper insight into the real adoption of AI chatbots in higher education, it is imperative to focus on measuring actual user behavior rather than solely relying on attitudinal or intention-based models, as the former approach provides a more accurate representation of the technology's integration within the educational context. Importantly, examining the temporal dynamics of usage behavior are essential for capturing nuanced alterations and complex effects related to ChatGPT adoption within higher education. As demonstrated in previous research (Croes and Antheunis, 2021; Skjuve et al., 2022), the temporal dynamics of technology usage can reveal valuable insights into the evolving relationship between users and AI chatbots, especially within the nascent stages of AI innovation. By investigating usage behavior changes over time, we can gain a deeper understanding of the adaptability and evolving needs of students as they engage with AI chatbots. Such insights can guide educational institutions and AI developers in tailoring their strategies and support systems to better accommodate the changing expectations of students in their AI adoption journey.

This study addresses a well-defined problem that lies at the intersection of technology acceptance, and higher education, exploring the adoption of ChatGPT by students. In this context, recent studies (Rudolph et al., 2023; Shoufan, 2023; Singh et al., 2023) have already provided initial findings on students' perceptions, beliefs and functionalities of ChatGPT, offering foundational insights into its application in educational contexts. Yet, it is essential to consider the temporal dynamics of such adoption due to the evolving nature of interactions with emerging AI chatbots. This temporal aspect, often overlooked in technology adoption research, allows for a more comprehensive understanding of how students continuously adopt, or reject, artificial intelligence innovations. Nonetheless, conventional technology acceptance theories, such as the Unified Theory of Acceptance and Use of Technology (UTAUT, Venkatesh et al., 2003, 2012) or its meta-analytical evolution (meta-UTAUT; Dwivedi et al., 2019) do not explicitly emphasize the temporal dynamics of technology adoption. These models provide valuable insights into the factors influencing adoption, but there is a gap in understanding the evolving relationship over time between users and AI systems such as ChatGPT. The technology adoption process is a continuous process where users' experiences, beliefs, and, eventually, behaviors change (Venkatesh and Davis, 2000; Wang et al., 2022). Therefore, it is of paramount importance to examine the progress as well as the drivers of adoption throughout the entire innovation life cycle (Rogers, 2003). Accordingly, in the context of ChatGPT, understanding how students' actual usage behavior evolves is critical for educational institutions and developers to adapt their policies and optimize the student-to-AI interactions in higher education.

Exploring the drivers of potential changes in ChatGPT usage behavior over time presents a significant challenge. First, while previously established factors of models of technology acceptance such as perceived ease of use and social influence (Venkatesh et al., 2003; Dwivedi et al., 2019) have been cornerstones of technology acceptance research, it is crucial to recognize that these factors often play a role as direct predictors of attitudes and intentions rather than direct indicators of actual behavior (Venkatesh et al., 2012). Also, despite users' behavioral intention been considered as a primary predictor of technology usage behavior, empirical evidence reveals only a low-to-medium effect size for this association (Bhattacherjee and Sanford, 2009). However, the limitations of these factors extend beyond their predictive value. Many of these constructs often remain relatively stable over time, making it less apparent how fluctuations in these factors can predict changes in usage behavior. Perceived ease of use (Davis, 1989), for instance, often pertains to the inherent ease of interacting with a technology and is unlikely to undergo significant shifts when it comes to students' interactions with ChatGPT. Also, while facilitating conditions have been proposed to as a key construct in the established technology acceptance models, their effect on students' desires to use ChatGPT was recently found to be non-significant (Strzelecki, 2023). Given these considerations, focusing on such factors as indicators of temporal changes in usage behavior can offer limited explanatory power or may not capture emerging constructs.

To address these research gaps, this brief research report employs a longitudinal design and emphasizes the unique role of specific psychological factors in understanding the potential change in students' ChatGPT usage behavior over time (i.e., between an initial phase T1 and a follow-up phase T2 8 months later). Such factors, namely trust, emotional creepiness, and Perceived Behavioral Control, were prudently selected as prominent factors with a recorded impact on technology adoption. Their selection for this study was based not only on their prominence in existing academic literature (e.g., Patil et al., 2020; Rajaobelina et al., 2021; Kelly et al., 2022) as drivers of acceptance but also on their potential for considerable fluctuation over time. Such variability makes them apt candidates for investigating the dynamic nature of student usage behavior with AI technologies such as ChatGPT.

In specific, trust is considered a crucial factor in technology acceptance confirming previous studies in other contexts (Glikson and Woolley, 2020; Patil et al., 2020; Choudhury and Shamszare, 2023), encapsulating a deeper bond between users and technology that evolves with continued interaction and experience. Trust is a dynamic construct that can be influenced by ongoing interactions and experiences (Wang and Siau, 2018), and fluctuations in this factor can significantly affect user behavior (Raffaghelli et al., 2022). Trust is inherently dependent on the reliability of ChatGPT output; if the AI system provides, for instance, vague or incorrect responses or exhibits inconsistent performance or reduced functionalities for students, trust may decline.

Furthermore, although emotions play a pivotal role in shaping users' acceptance and interaction with technology (Venkatesh, 2000; Lu et al., 2019), their effects in the context of students' adoption of ChatGPT remain largely underexplored. Emotional creepiness, a relatively unexplored yet impactful factor (Langer and König, 2018; Rajaobelina et al., 2021), brings to the forefront the affective responses that can significantly deter or encourage technology adoption, particularly in AI where human-like interactions are prevalent. Students' experience of emotional creepiness may diminish over time as students become more accustomed to the technology through adaptive responding and emotion regulation (Kashdan et al., 2015). In a similar vein, Perceived Behavioral Control, a well-established construct in the Theory of Planned Behavior with a direct impact on human behavior (Ajzen, 1991) may fluctuate over time due to evolving policies, institutional restrictions, and changing guidelines that impact students' interaction with ChatGPT. Hence, the examination of these factors in the context of students' use of ChatGPT seems promising for providing insights into the changing landscape of AI adoption in higher education.

Also, building on the insights from Rabardel and Bourmaud (2003), Carroll (2004), and Kaptelinin and Nardi (2006), this study considers the concept of “instrumental genesis” and the processes of “instrumentalization” and “instrumentation” in the context of AI chatbots in higher education. These perspectives emphasize the dynamic and evolving nature of technology use, where students actively shape and reconfigure their interactions with ChatGPT over time, contributing to the continuous development of their usage patterns.

The present research report contributes to the existing body of knowledge by (a) shedding light on the dynamic nature of students' AI chatbot adoption in higher education, examining if students' usage behavior of ChatGPT changes over time (i.e., between the initial and follow-up phases T1 and T2), and (b) exploring the factors (i.e., trust, emotional creepiness, Perceived Behavioral Control) that underlie these changes, by examining if changes such factors over time can accurately predict fluctuations in students' usage behavior of ChatGPT.

## 2 Theoretical background

### 2.1 Navigating the web of trust

Trust in AI, a pivotal component of human-computer interaction, has garnered increasing attention in recent years due to the proliferation of AI-driven technologies across various domains (Glikson and Woolley, 2020). Trust, as a psychological factor, plays a crucial role in users' willingness to engage with and rely on IT/IS systems such as chatbots (Følstad et al., 2018). It is an essential psychological construct that significantly influences human-AI relationships, shaping the way individuals perceive, interact with, and ultimately utilize AI intelligent systems. The importance of trust in AI is highlighted by the critical role it plays in users' acceptance, adoption, and continued use of AI technologies. Empirical evidence that trust in AI is intricately linked to user behavior, with higher levels of trust leading to more positive attitudes and thus to greater use behavior (Patil et al., 2020; Raffaghelli et al., 2022). Users tend to engage with AI technologies they perceive as trustworthy, reliable, and transparent (Dwivedi et al., 2021). While transparent AI systems can instill confidence in users, opaque AI systems may lead to user skepticism and hinder actual usage behavior of AI technologies. Interestingly, researchers have recognized that trust is a dynamic concept (McKnight et al., 2002; Lee and Choi, 2011), as it progressively evolves over time through the repetition of an action (Gefen et al., 2003). Thus, user experiences can modify trust, and there is actually a repeated loop of trust–action–learning–trust (Urban et al., 2009; Lin et al., 2014).

Within the realm of higher education, trust in AI technologies, including AI chatbots like ChatGPT, is crucial for shaping students' academic experiences and their adoption of these technologies. ChatGPT can primarily assist by retrieving and summarizing information in response to student queries. While it may not offer advanced personalization through a sophisticated user model, its ability to provide concise information tailored to specific queries is where its utility lies for students. In this context, students' trust in the accuracy, reliability, and usefulness of ChatGPT's responses becomes a pivotal factor influencing their usage and engagement with the technology. On the other hand, a decline in trust, perhaps due to experiences with inaccurate or inconsistent responses from ChatGPT, could lead to decreased reliance on and engagement with the chatbot. Thus, understanding the nuances of how trust in ChatGPT develops and changes over time is essential for comprehending its adoption and sustained use in educational settings. However, while recent research has identified trust as a key driver of ChatGPT usage behavior in various contexts, such as healthcare (Choudhury and Shamszare, 2023), its specific impact within the realm of higher education has yet to be empirically investigated.

Furthermore, the concept of “instrumental genesis” as described by Kaptelinin and Nardi (2006) aligns with this dynamic understanding of trust. It suggests that students' trust in ChatGPT may evolve as they adapt the technology to their specific academic needs and contexts, reflecting a developmental perspective on trust formation. Therefore, exploring the dynamic nature of trust in AI within the higher education context is of paramount importance for understanding the evolving nature of student adoption of ChatGPT.

### 2.2 The human-AI emotional interplay and emotional creepiness

The effects of experienced emotions on subsequent judgement and decision-making has been well-established (Lerner et al., 2015). Although the influence of emotions is critical in determining how users accept and interact with technology (e.g., Saadé and Kira, 2006; Lu et al., 2019), the impact of specific emotional factors on students' adoption of ChatGPT has not been studied. Thus, delving into this less explored area is crucial for enriching our understanding of AI integration in higher education.

In the context of AI adoption, emotions emerge as an influential factor that shapes individuals' interactions with technology (Rapp et al., 2021). Among these emotions, the prominence of negative feelings cannot be understated, as, for instance, unease, discomfort, or even aversion, can significantly mold the dynamics of AI adoption (Skjuve et al., 2019). Likewise, when students experience negative emotions in their interactions with AI chatbots, such emotions can hinder their usage behavior.

Emotional experiences encompass various aspects, but one that holds particular significance is the emerging concept of “emotional creepiness” (Langer and König, 2018). In general, people tend to refer to ambiguous situations, or ones that evoke uneasy feelings as “creepy.” The experience of emotional creepiness may be associated with states of emotional uncertainty, which has been shown to shape subsequent judgments (Polyportis et al., 2020). In the context of AI adoption, emotional creepiness describes the unsettling emotions or discomfort that individuals may encounter when interacting with AI systems that mimic human emotions or intentions. Emotional creepiness thus relates to the delicate balance between AI that provides a user-friendly experience and AI that elicits disconcerting or eerie interactions (Langer and König, 2018; Rajaobelina et al., 2021).

The concept of emotional creepiness can arise in response to AI systems that cross ethical or privacy boundaries, make inappropriate recommendations, or even exhibit unpredictable behaviors. What adds another layer of complexity to the understanding of emotional creepiness is its dynamic nature. With prolonged exposure and continuous human-AI interactions, users may find that their negative emotional responses gradually evolve via adaptive responding and negative emotion regulation (Kashdan et al., 2015). Therefore, emotional creepiness might diminish over time as individuals become more accustomed to the AI system interactions and gain familiarity with its characteristics. As students interact with ChatGPT over time, it is plausible that their initial perceptions of creepiness may give way to a more normalized acceptance. This improved emotional experience, in turn, can significantly influence subsequent changes in students' usage behavior of ChatGPT. The evolution of emotional responses, particularly emotional creepiness, can also be understood through the lens of “instrumentalization” and “instrumentation” (Rabardel and Bourmaud, 2003). As students interact more with ChatGPT, they may reconfigure their emotional responses based on their developing understanding and familiarity with the AI, leading to changes in their perceptions and usage behavior.

### 2.3 Perceived Behavioral Control and AI adoption

Perceived Behavioral Control (PBC) is a fundamental psychological construct with a rich history in the study of human behavior. Derived from the Theory of Planned Behavior (Ajzen, 1991), Perceived Behavioral Control represents an individual's subjective assessment of their capability to perform a specific behavior. It encapsulates factors such as personal skills, resource availability, and external constraints, and is intimately linked to the notion of self-efficacy (Bandura, 1977). In essence, Perceived Behavioral Control serves as a determinant of an individual's ability to enact a behavior. The higher the level of Perceived Behavioral Control, the more likely a person is to engage in the intended action (Ajzen, 2002). Nonetheless, previous research has put forward that any effects of Perceived Behavioral Control in technology acceptance are often overlooked (Zolait, 2014).

Perceived Behavioral Control is known to directly influence human behavior (Ajzen, 1991), it has yet to be extensively studied in the context of its effects on ChatGPT usage behavior, marking an important area for research exploration. Perceived Behavioral Control can play a pivotal role in AI and technology adoption, as it can significantly influence usage intentions and behavior (Taylor and Todd, 1995; Kelly et al., 2022). Accordingly, students' engagement with AI systems and their decision to incorporate AI technologies into their educational experiences is influenced by their Perceived Behavioral Control. The presence of institutional policies and guidelines can significantly impact students' sense of control over their interactions with emerging AI systems, such as ChatGPT. In specific, the development and implementation of policies and the evolution of institutional restrictions may introduce alterations in students' Perceived Behavioral Control over time, leading to adjustments or decrease of their usage behavior. Thus, by delving into the dynamic relationship between Perceived Behavioral Control and students' AI adoption, we can achieve a more nuanced understanding of students' AI acceptance in the realm of higher education.

Carroll's (2004) notion of “completing design in use” also resonates with the concept of Perceived Behavioral Control. As students navigate and adapt to the evolving AI landscape in higher education, they play an active role in shaping how they use and control their interactions with ChatGPT, reflecting a continuous cycle of adaptation and appropriation.

To summarize, the focal point of investigation pertains to whether changes of trust, emotional creepiness and Perceived Behavioral Control can significant predict student's usage behavior change of ChatGPT. Stated more formally:

Δ*Y*i = f(Δ*X*i) + ε, where:

Δ*Y*i= students' usage behavior change of ChatGPT.

Δ*X*i= changes in trust, emotional creepiness, Perceived Behavioral Control.

Δ denotes the change in each variable over time (from phase T1 to T2).

## 3 Materials and methods

### 3.1 Data collection

In this study, a two-phase survey approach was employed, targeting students in Dutch higher education institutions. Within Phase 1, the research commenced with the distribution of a questionnaire to 420 participants through the online Prolific platform. To guarantee the eligibility of the respondents, stringent pre-screening criteria were applied, necessitating that all participants were students currently enrolled in academic institutions in the Netherlands. To reinforce the stringency of the process, a question was incorporated, requiring participants to answer with a “yes” or “no” to confirm their current enrollment status as students in Dutch academic institutions. Also, given that the questionnaire included questions related to students' usage behavior of ChatGPT in higher education, it was imperative to collect a sample of students that had a previous experience with the use of the specific AI tool. In this respect, a screening question was included to identify a representative subset of students who were acquainted with and had utilized ChatGPT in their educational activities among the initial 420 participants. Among these, 355 participants responded positively, thereby meeting the eligibility criteria for the study.

Phase 2 of the study was carried out 8 months later by dispatching customized invitations to the initial 355 respondents. Within a week, 244 responses were received (response rate 67.6%). In the interest of ensuring that the respondents were still students enrolled in higher education, a screening question was introduced, resulting in the exclusion of twenty-two responses. Consequently, a final dataset of 222 respondents (M_age_ = 22.82 years, SD_age_ = 4.148, 43% females) who were students in Dutch higher education institutions and had provided complete responses during both phases of the survey, was retained for the subsequent analyses.

### 3.2 Instrument and measures

To optimize the content validity of the measures, established scales developed by other scholars were used and adapted to the context of this study. The structure of the questionnaires of both phases was identical. Each questionnaire initially included measures of students' usage behavior of ChatGPT in their educational activities (four items adjusted from Patil et al., 2020). Afterwards, the questionnaire consisted of measures of perceived trust (three items adjusted from Patil et al., 2020), emotional creepiness (four items adjusted from Langer and König, 2018) and Perceived Behavioral Control (three items from Taylor and Todd, 1995). Finally, participants responded to demographics and were thanked for their participation. All items were measured in seven-point (1: “Strongly disagree” – 7: “Strongly agree”) Likert scales. Detailed information on the measurement items of the abovementioned constructs are given in Table 1.

**Table 1:** Constructs and measures.

**Construct**	**Items**	**Source**
Trust	I trust ChatGPT to be reliable.	Patil et al., 2020
	I trust ChatGPT to be secure.	
	I trust ChatGPT to be trustworthy.	
Emotional creepiness	When using ChatGPT as a student I feel uneasy.	Langer and König, 2018
	When using ChatGPT I have an indefinable fear.	
	When using ChatGPT I have a queasy feeling.	
	When using ChatGPT I somehow feel threatened.	
Perceived Behavioral Control	I would be able to use ChatGPT as a student.	Taylor and Todd, 1995
	Using ChatGPT as a student is entirely within my control.	
	I have the resources *and* the knowledge *and* the ability to make use of ChatGPT as a student.	
Usage behavior	I use ChatGPT as a student.	Patil et al., 2020
	I use ChatGPT for my learning activities.	
	I use ChatGPT to fulfill my academic responsibilities.	
	I use ChatGPT to perform my academic assignments.	

### 3.3 Results

#### 3.3.1 Exploratory factor analysis

An exploratory factor analysis was conducted for both Phase 1 and Phase 2 to ascertain the common factors among the scale items used in the study and to assess the variables' validity. Principal component analysis with varimax rotation was employed for factor extraction, considering Eigen values equal to or >1. The analysis successfully extracted all anticipated variables in both phases. For Phase 1, the Bartlett test of sphericity yielded a significant result of 2772.36 (*p* < 0.001), and the Kaiser–Meyer–Olkin statistic recorded a value of 0.83 (>0.6), confirming the suitability of the data for identifying factor dimensions. For Phase 2, the Bartlett test of sphericity yielded a significant result of 2249.45 (*p* < 0.001), and the Kaiser–Meyer–Olkin statistic recorded a value of 0.82 (>0.6), confirming the suitability of the data for identifying factor dimensions. To assess reliability, Cronbach's α was used. As presented in Tables 2, 3 below, all variables demonstrated scale validity and reliability, affirming their suitability for the final analysis of the study.

**Table 2:** Factor analysis, Phase 1.

**Factor**	**Component**			
	**1**	**2**	**3**	**4**
Usage behavior	0.917			
	0.904			
	0.820			
	0.787			
Trust		0.924		
		0.893		
		0.881		
Emotional creepiness			0.873	
			0.853	
			0.832	
			0.800	
Perceived Behavioral Control				0.912
				0.896
				0.877
Cronbach's α	0.94	0.92	0.88	0.95
KOM	0.83			
Bartlett's test of sphericity	2772.36^***^			

^***^p < 0.001.

**Table 3:** Factor analysis, Phase 2.

**Factor**	**Component**			
	**1**	**2**	**3**	**4**
Usage behavior	0.882			
	0.844			
	0.837			
	0.835			
Trust		0.823		
		0.804		
		0.789		
Emotional creepiness			0.866	
			0.851	
			0.847	
			0.714	
Perceived Behavioral Control				0.961
				0.948
				0.935
Cronbach's α	0.91	0.84	0.86	0.95
KOM	0.82			
Bartlett's test of sphericity	2249.45^***^			

^***^p < 0.001.

#### 3.3.2 Preliminary multiple regression analysis, phase T1

Age and gender were included as control variables to account for potential demographic influences on usage behavior (Venkatesh and Morris, 2000; Rogers, 2003), ensuring a more comprehensive analysis. The results of a preliminary multiple regression analysis at phase T1 with usage behavior as a dependent variable, and trust, emotional creepiness, and Perceived Behavioral Control at T1, together with age and gender, as independent variables revealed a statistically significant model, *F*_(5, 216)_ = 26.276, *p* < 0.001, indicating that the combined set of predictors significantly explained the variance in usage behavior at T1. The model's R-squared value was 0.378, demonstrating that 37.8% of the variance in T2 usage behavior was accounted for by the predictors. Trust had a significant positive effect (β = 0.193, *p* < 0.001), implying that higher levels of trust at T1 were associated with increased usage behavior at T1. Conversely, emotional creepiness had a significant negative effect (β = −0.127, *p* < 0.05), suggesting that higher levels of emotional creepiness at T1 were linked to decreased usage behavior at T1. Perceived behavioral control (PBC) had a significant positive influence (β = 0.436, *p* < 0.001), indicating that greater Perceived Behavioral Control at T1 was associated with increased usage behavior at T1. Age and gender, however, did not significantly predict usage behavior at T1 (*p* > 0.05). These results provide support for the notion that trust, emotional creepiness, and Perceived Behavioral Control significantly predict usage behavior at T1.

#### 3.3.3 Temporal changes in usage behavior and key constructs between phases T1 and T2

The correlation between students' usage behavior of ChatGPT at Phases T1 and T2 was statistically significant, *r* = −0.250, *p* < 0.01. To examine how students' usage behavior of ChatGPT changed from T1 to T2, a paired-samples t-test was conducted. The analysis comparing the means of usage behavior at T1 (M = 3.67, SD = 1.82) and T2 (M = 3.05, SD = 1.53) revealed a significant difference in student' usage behavior of ChatGPT, *t*_(221)_ = 3.48, *p* = 0.001, M_diff._ = −0.62, 95% CI [0.27, 0.97]. Hence, a significant reduction in students' usage behavior of ChatGPT became evident.

In a similar vein, a paired-samples *t*-test was conducted to compare trust scores between T1 (M = 3.84, SD = 1.55) and T2 (M = 3.08, SD = 1.38). The results revealed a statistically significant difference in trust scores over time, *t*_(221)_ = 5.14, *p* < 0.001, with trust scores decreasing from T1 to T2 [M_diff._ = −0.76, 95% CI (0.47, 1.05)]. In addition, a paired samples *t*-test was performed to compare Perceived Behavioral Control (PBC) scores between T1 (M = 4.93, SD = 1.59) and T2 (M = 3.50, SD = 1.66). The results revealed a statistically significant difference in PBC scores over time, *t*_(221)_ = 11.36, *p* < 0.001, with PBC scores decreasing from T1 to T2 [M_diff._ = −1.43, 95% CI (1.18, 1.68)]. Likewise, a paired samples *t*-test was conducted to compare emotional creepiness scores between T1 (M = 2.81, SD = 1.38) and T2 (M = 2.34, SD = 1.28). The results unveiled a statistically significant difference in creepiness scores between T1 and T2, *t*_(221)_ = 4.65, *p* < 0.001, indicating a decrease in emotional creepiness from T1 to T2 [M_diff._ = −0.46, 95% CI (0.27, 0.66)]. Table 4 below includes the descriptive statistics for the key constructs.

**Table 4:** Descriptive Statistics for ChatGPT usage behavior, trust, Perceived Behavioral Control, and emotional creepiness at T1 and T2.

**Construct**	**Mean T1**	**SD T1**	**Mean T2**	**SD T2**
Usage behavior	3.67	1.82	3.05	1.53
Trust	3.84	1.55	3.08	1.38
Perceived Behavioral Control	4.93	1.59	3.50	1.66
Emotional creepiness	2.81	1.38	2.34	1.28

#### 3.3.4 Analysis to predict students' ChatGPT usage behavior change

To examine whether changes in trust, emotional creepiness and Perceived Behavioral Control can significantly predict changes in students' usage behavior of ChatGPT, latent variables denoted as ΔXi were constructed for each respondent, representing the change in scores each of the independent variables, where Δ equals the score of each variable at T2 minus the score at T1. The same procedure was applied to the dependent variable (usage behavior) to assess changes denoted as ΔYi in students' usage behavior over time.

**Figure 1:**
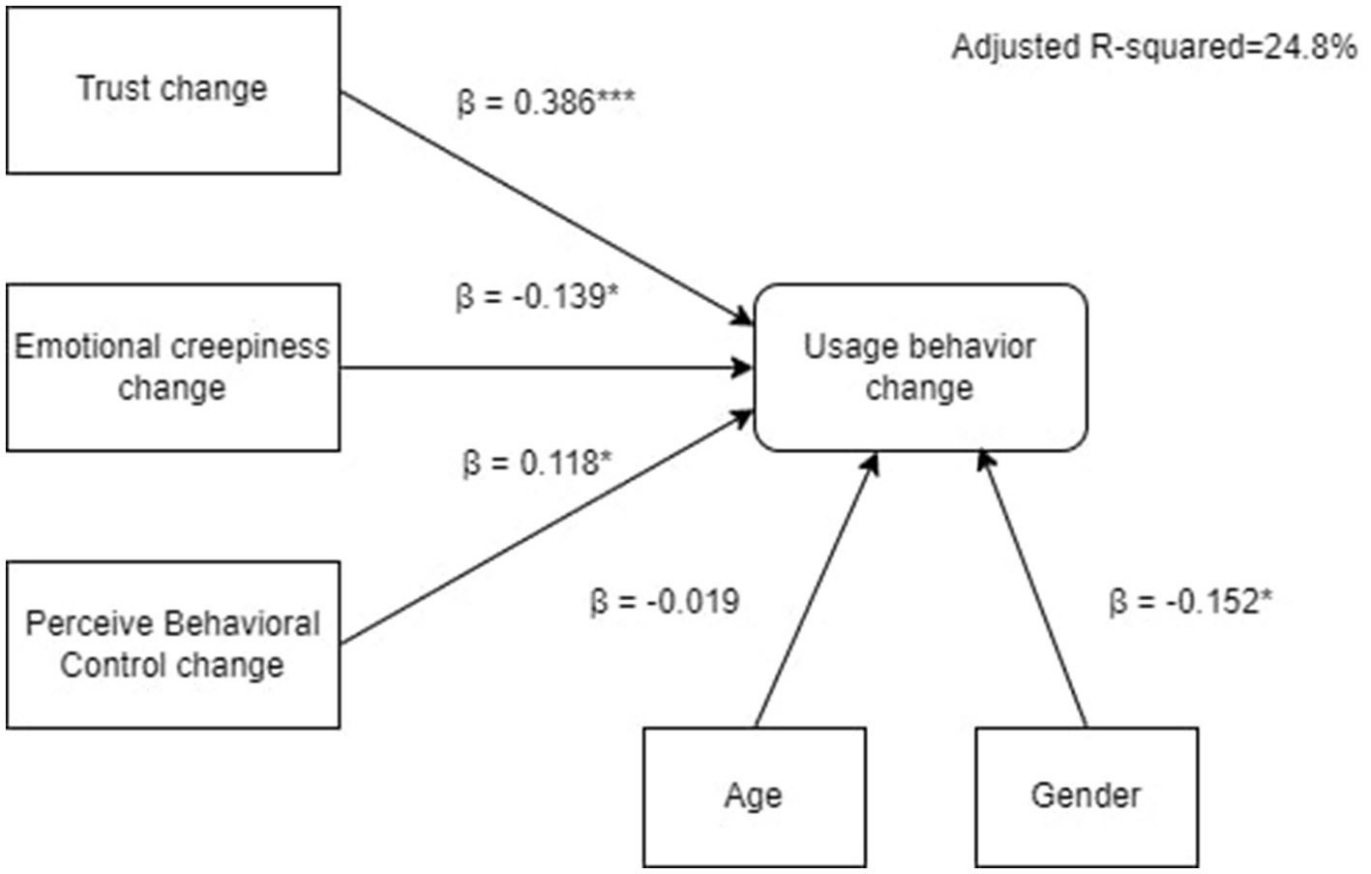
Antecedents of students' ChatGPT usage behavior change. *p < 0.05, ***p < 0.001.

A multiple regression analysis was afterwards conducted with usage behavior change as the dependent variable, and changes in trust, emotional creepiness, Perceived Behavioral Control, together with age and gender, as independent variables. The analysis sought to explore the drivers of students' usage behavior change of ChatGPT. The regression model was statistically significant, *F*_(5, 216)_ = 15.602, *p* < 0.001, demonstrating an adjusted R-squared of 0.248, suggesting that 24.8% of the variability in usage behavior change was explained by the combination of these factors. The coefficients of the predictors of usage behavior change were as follows: Trust change [β = 0.386, *p* < 0.001, 95% CI (0.317, 0.613)], emotional creepiness change [β = −0.139, *p* < 0.05, 95% CI (-0.467,−0.032)], Perceived Behavioral Control change [β = 0.118, *p* < 0.05, 95% CI (0.004, 0.331)], age [β = −0.019, *p* >0.05, 95% CI (-0.086, 0.062)], and gender [β = −0.152, *p* < 0.05, 95% CI (-1.444,−0.188)] (see Figure 1).

## 4 Discussion

### 4.1 Theoretical and practical implications

The present study sought to investigate if and how students' usage behavior with ChatGPT changes over time and to explore the drivers of students' usage behavior change. This approach was rooted in recognizing the dynamic nature of the relationship between students and AI chatbots in the realm of higher education. The present findings not only shed light on the evolving landscape of AI adoption but also underscore the implications these shifts have for academia and educational institutions (Dwivedi et al., 2023). The longitudinal study revealed a notable decrease in ChatGPT usage from the initial phase (T1) to a subsequent phase (T2).

The findings of this study, indicating the crucial role of trust, emotional creepiness, and Perceived Behavioral Control in predicting changes in students' ChatGPT usage behavior, offer a nuanced understanding of technology adoption in educational settings. The positive regression coefficients for trust and Perceived Behavioral Control indicate that increases in these factors are associated with an increase in usage behavior. This aligns with the broader technology acceptance literature, which posits that users' trust in a technology's reliability and effectiveness (Venkatesh, 2000; Gefen et al., 2003) and their perceived control over its use (Ajzen, 1991) are critical determinants of its adoption. Trust, in particular, emerges as a dynamic construct, continually shaped by users' interactions with and experiences of the technology (Patil et al., 2020). The findings support the notion that enhancements in trust and PBC significantly contribute to positive changes in students' usage of ChatGPT.

Interestingly, the measures of trust and Perceived Behavioral Control at T1 and T2 revealed a decline over time. The decrease in trust may be attributed to growing concerns about the reliability of ChatGPT's output or to wider public discussions, while the reduction in Perceived Behavioral Control could stem from the introduction of new policies and regulations within Dutch higher education institutions.

It is also important to acknowledge the role of emotional creepiness as an emerging factor in understanding artificial intelligence acceptance. The observed decrease in emotional creepiness, associated with increased familiarity and comfort, underscores the importance of the affective aspects of technology interaction (Langer and König, 2018; Rajaobelina et al., 2021), which are often overlooked. A decrease in emotional creepiness was found to lead to a subsequent increase in usage behavior. This aspect emphasizes the need for user-friendly design and ethical considerations within AI development (Ryan, 2020). AI technologies that are perceived as intrusive or ethically ambiguous may heighten feelings of emotional creepiness, thereby hindering their acceptance.

From a practical standpoint, these insights have significant implications for educational institutions, which may consider adopting an adaptive approach in their strategies, considering the evolving nature of student-chatbot interactions. This may include continuous monitoring, evaluation, and refinement of chatbot functionality to meet changing student needs. Moreover, institutions should invest in resources to ensure the consistent and reliable performance of AI chatbots, as this directly impacts students' trust and, consequently, their usage behavior. Educational policymakers should establish and communicate clear policies and guidelines for AI chatbot usage within the educational context. The significant role of Perceived Behavioral Control in predicting changes in usage behavior underscores the need for supportive institutional environments. This involves addressing factors like policies, privacy concerns, and ethical considerations (Kooli, 2023).

### 4.2 Limitations and future research

This study does not come without limitations. Firstly, the reliance on self-reported data raises concerns about the depth of the insights gathered. While self-reporting is a common and practical method in survey research, it inherently relies on participants' perceptions, which may not fully capture the complexity of their interactions with ChatGPT. Secondly, the study's methodology, involving only two measurement points, offers a limited temporal perspective. This design may provide a valuable snapshot of usage behavior changes but does not capture the continuous evolution of this behavior over time. The dynamism of artificial intelligence adoption, particularly in rapidly changing educational environments, calls for more frequent observations to accurately track and understand these changes.

Additionally, this study did not delve into the specific details of ChatGPT usage among students, such as the frequency of use and the diverse ways in which ChatGPT was employed for academic purposes. This omission leaves a gap in our understanding of how students are integrating ChatGPT into their learning processes and the extent to which it influences their academic experiences. These limitations underscore the need for future research with more granular data collection methods, including open-ended questions and mixed-method approaches, to enrich the understanding of AI adoption in educational contexts. Future studies should aim to address these gaps, possibly by incorporating longitudinal designs with multiple data collection points and detailed inquiries into the specific usage patterns of AI tools like ChatGPT. This approach would provide a more comprehensive and accurate portrayal of the evolving relationship between students and AI technologies in higher education.

In conclusion, this study provides insights into the evolving dynamics of students' usage behavior with ChatGPT in higher education. The observed temporal changes in usage behavior emphasize the need for adaptability and responsive strategies. Furthermore, by considering trust, emotional creepiness, and Perceived Behavioral Control, educational institutions can ensure that responsible AI systems (Dignum, 2021; Polyportis and Pahos, In press) can facilitate enhancing the learning experience, and eventually fostering a more dynamic and effective learning environment.

## Data availability statement

The raw data supporting the conclusions of this article will be made available by the authors, without undue reservation.

## Ethics statement

The studies involving humans were approved by the Human Research Ethics Committee of Delft University of Technology. The studies were conducted in accordance with the local legislation and institutional requirements. The participants provided their written informed consent to participate in this study.

## Author contributions

AP: Conceptualization, Investigation, Methodology, Resources, Visualization, Writing—original draft, Writing—review & editing.
